# Draft Genome Sequence of a Novel Adenovirus Recovered from the Metagenome of Agile Wallabies

**DOI:** 10.1128/mra.00112-23

**Published:** 2023-05-18

**Authors:** God’spower Richard Okoh, Ellen Ariel, David Whitmore, Paul F. Horwood

**Affiliations:** a James Cook University, College of Public Health, Medical and Veterinary Sciences, Townsville, Queensland, Australia; Katholieke Universiteit Leuven

## Abstract

Here, we report the draft genome sequence of a novel agile wallaby adenovirus that was detected in the fecal metagenome of agile wallabies. The genome is 31,512 bp long, with a G+C content of 34.4%. Currently, the pathogenic and zoonotic potential of this novel virus is unknown.

## ANNOUNCEMENT

Adenoviruses are nonenveloped, icosahedral viruses with linear, unsegmented double-stranded DNA genomes (1). The genomes of adenoviruses range in size from 26 to 48 kb and commonly contain between 22 and 40 genes (1, 2). The family *Adenoviridae* is divided into six genera, namely, *Mastadenovirus*, *Aviadenovirus*, *Atadenovirus*, *Siadenovirus*, *Ichtadenovirus*, and *Testadenovirus* (1, 3). Adenoviral infections do not always result in disease, although they have been associated with both single and multipathogen disease processes (4–8).

The novel Agile wallaby atadenovirus 1 (AwAdV-1) described in this report belongs to the genus *Atadenovirus* and was originally identified in the metagenome of free-ranging agile wallabies (Notamacropus agilis) (G. R. Okoh, E. Ariel, D. Whitmore, P. F. Horwood, submitted for publication). Briefly, five fresh fecal samples were collected from the ground at grazing sites around James Cook University and Townsville University Hospital (Townsville, Australia) in 2021. The samples were homogenized, pooled, and then virally enriched by filtration (0.25 μm), ultracentrifugation (100,000 × *g*), and digestion with DNase I (20 U/mL). Viral DNA was then extracted using QIAamp MinElute virus kit (Qiagen). Library preparation using the Nextera DNA XT kit and Illumina sequencing (NovaSeq 6000) were performed at Macrogen (Seoul, South Korea) in paired-end 151-bp format. For this report, the sequencing reads (71,170,820) were trimmed (Trimmomatic v0.39) (9) to remove low-quality reads, normalized using BBNorm v39.01 (https://sourceforge.net/projects/bbmap/), and *de novo* assembled using SPAdes v3.15.5 in “careful” mode (10). The resulting contigs were searched using Diamond BLASTX (11) against the NCBI nonredundant (nr) protein database to identify the contigs corresponding to adenovirus. To assemble the genome, the reads were mapped to the identified adenoviral contig using Geneious v11.1.5 (https://www.geneious.com). Prediction of open reading frames (ORFs) was performed using Glimmer3 in Geneious v11.1.5, and ORF annotations were determined by conducting a BLASTX search against the NCBI nr protein databases (12). All tools were run with default parameters unless otherwise specified.

**FIG 1:**
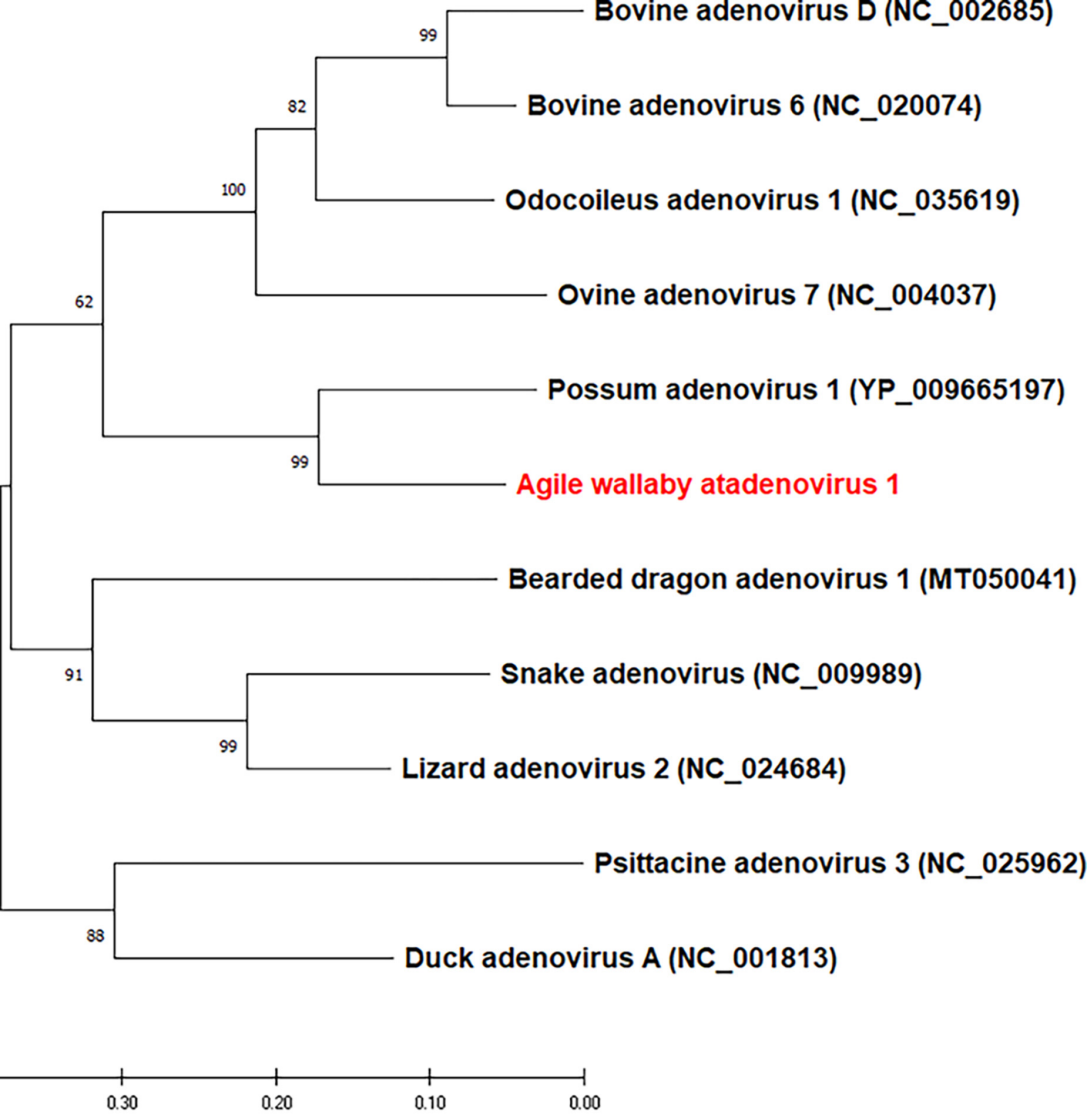
Phylogenetic analysis of Agile wallaby atadenovirus 1 (shown in red), based on the amino acid sequence of penton-based protein. All the sequences used in this analysis belong to the genus *Atadenovirus*. Following multiple sequence alignment of amino acid sequences using the Muscle program in Geneious v11.1.5, a maximum likelihood phylogenetic tree was constructed using MEGA X (13) with the best model of amino acid substitution (LG + G) and 1,000 bootstrap replications.

The assembled genome of AwAdV-1 was found to be 31,512 bp long, with a coverage depth of 22× and 34.4% G+C content. The genome was predicted to contain 32 ORFs with an orientation typical of atadenoviruses. Of the 32 ORFs, 26 were annotated as having various similarities to the coding genes of other atadenoviruses (Tables 1). The IVa2, penton base protein, PX, and hexon genes showed the highest amino acid identity (71% to 87%) to the reference mammalian atadenoviruses (Table 1). The AwAdV-1 genome possesses multiple insertions and deletions in most of the genes except IVa2, pX, pVI, pVIII, and U-exon. Two fiber genes, namely, fiber and IV-1 (homologous to the fiber 2 gene in lizard adenovirus 2), were present in the genome of AWAdV-1, instead of the single long fiber gene in mammalian atadenoviruses. Phylogenetic analysis based on the full amino acid sequence of penton base protein showed that AwAdV-1 belongs to the genus *Atadenovirus* and forms a distinct cluster with another marsupial adenovirus known as possum adenovirus 1 (Fig. 1).

**TABLE 1:** Sequence comparison with the genomes of mammalian atadenoviruses

ORF	Description (putative)	Data for virus (GenBank accession no.):^*a*^													
		Agile wallaby atadenovirus 1		*Ovine adenovirus* (NC_004037)			*Bovine adenovirus D* (NC_002685)			*Bovine adenovirus E* (NC_020074)			*Odocoileus adenovirus 1* (NC_035619)		
		Length (nt)	Length (aa)	Length (nt)	Length (aa)	Amino acid identity (%)	Length (nt)	Length (aa)	Amino acid identity (%)	Length (nt)	Length (aa)	Amino acid identity (%)	Length (nt)	Length (aa)	Amino acid identity (%)
orf0001	Hypothetical protein	255	85	No data	No data	No data	No data	No data	No data	No data	No data	No data	No data	No data	No data
orf0002	Hypothetical protein	402	134	No data	No data	No data	No data	No data	No data	No data	No data	No data	No data	No data	No data
orf00003	p32K	1,032	344	861	286	39	819	272	45	891	296	40	930	309	35
orf00004	LH1	396	132	363	120	29	378	125	29	378	125	26	393	130	25
orf00005	E1B 55K	1,293	431	1149	382	43	1,161	386	42	1,152	383	45	1,146	381	43
orf00006	IVa2	903	301	984	327	75	966	321	74	966	321	72	1,209	402	72
orf00007	Pol	3,243	1,081	3,216	1,071	58	3,222	1,073	58	3,222	1,073	57	3,228	1,075	58
orf00008	pTP	1,773	591	1,788	595	50	1,803	600	51	1,803	600	51	1,800	599	51
orf00009	52K	987	329	1,008	335	62	1,059	343	62	1,035	344	60	1,014	337	58
orf00010	pIIIa	1,767	589	1,707	568	53	1,722	573	54	1,551	516	54	1,749	582	53
orf00011	Penton base protein	1,347	449	1,359	452	68	1,353	450	67	1,359	452	68	1,353	450	71
orf00012	pVII	345	115	336	111	54	360	119	57	357	118	56	354	117	51
orf00013	pX	102	34	216	71	87	216	71	81	219	72	0	No data	No data	No data
orf00014	pVI	669	223	666	221	54	603	200	57	612	203	56	678	225	54
orf00015	Hexon	2,730	910	2,736	911	74	2,733	910	72	2,733	910	76	2,733	910	74
orf00016	23K endoprotease	606	202	606	201	59	606	201	60	606	201	61	606	201	60
orf00018	DNA binding protein	999	333	1,149	382	56	1,143	380	58	1,140	379	56	1,158	385	57
orf00020	100K	2,055	685	1,878	625	54	1,887	628	56	1,887	628	54	1,914	637	55
orf00021	33k	512	170	402	133	39	405	134	65	408	135	39	414	137	38
orf00022	pVIII	780	260	654	217	44	669	222	44	672	223	45	681	226	49
Orf00023	U-exon	165	55	177	58	35	165	54	54	165	54	54	165	54	54
orf00024	Fiber	891	297	1,632	543	36	1,608	535	36	1,332	443	43	1,422	473	29
orf00025	IV-1	1,554	518	No data	No data	No data	No data	No data	No data	No data	No data	No data	No data	No data	No data
orf00026	E4.3	600	200	714	237	34	654	217	38	657	218	38	705	234	33
orf00027	E4.2	678	226	663	220	40	678	219	42	660	219	42	678	219	39
orf00028	E4.1	441	147	429	142	36	429	142	37	429	142	38	429	142	30
orf00029	RH0	381	127	No data	No data	No data	564	187	45	No data	No data	No data	No data	No data	No data
orf00030	RH5	606	202	597	198	26	624	207	27	651	216	24	624	207	34
orf00031	Hypothetical protein	372	124	No data	No data	No data	No data	No data	No data	No data	No data	No data	No data	No data	No data
orf00032	Hypothetical protein	201	67	No data	No data	No data	No data	No data	No data	No data	No data	No data	No data	No data	No data
orf00033	Hypothetical protein	366	122	No data	No data	No data	No data	No data	No data	No data	No data	No data	No data	No data	No data
orf00034	Hypothetical protein	372	124	No data	No data	No data	No data	No data	No data	No data	No data	No data	No data	No data	No data

a aa, amino acids.

The pathogenic potential of AwAdV-1 is unclear; however, it could be a suitable candidate for future research in vaccinology, diagnostics, and therapeutics.

### Data availability.

The raw sequence reads for this study have been deposited in the NCBI SRA database under BioProject accession number PRJNA907146 and BioSample accession number SAMN31952915. The novel genome sequence has been deposited at GenBank under the accession number OQ792214.
